# Osmotin Protects H9c2 Cells from Simulated Ischemia-Reperfusion Injury through AdipoR1/PI3K/AKT Signaling Pathway

**DOI:** 10.3389/fphys.2017.00611

**Published:** 2017-09-25

**Authors:** Jianhua Liu, Hua Sui, Jianlin Zhao, Yan Wang

**Affiliations:** ^1^Department of Cardiology, Xinxiang Central Hospital Xinxiang, Henan, China; ^2^Department of Endocrinology, Xinxiang Central Hospital Xinxiang, Henan, China

**Keywords:** osmotin, acute myocardial infarction, ischemia-reperfusion injury, AdipoR1/PI3K signaling pathway, AKT signaling pathway

## Abstract

**Objective:** This study aimed to investigate the effect of osmotin on myocardial ischemia/reperfusion (I/R), as well as the underlying mechanisms.

**Methods:** *In vitro* I/R injury model was established on rat cardiac myoblast H9c2 cells by oxygen and glucose deprivation followed by reperfusion (OGD/R). Cells were administrated with osmotin, and transfected with small interfering RNAs (siRNAs) which specifically target adiponectin receptor 1 or 2 (AdipoR1/2). Besides, the cells were incubated with or without LY294002 as inhibitor of phosphatidylinositol 3-kinase (PI3K) under OGD/R condition. Cell viability, apoptosis, expressions of apoptosis-related proteins and inflammatory factors were analyzed.

**Results:** The results showed that osmotin significantly increased H9c2 cells viability compared with the cells treated with vehicle (*P* < 0.05), and decreased H9c2 cells apoptosis by regulating expressions of apoptosis-related proteins. Moreover, we observed that osmotin statistically reduced the release of proinflammatory factors and increased the release of anti-inflammatory factors in H9c2 cells (*P* < 0.05). However, these effects were markedly reversed by AdipoR1 silence but not AdipoR2. Furthermore, osmotin dramatically upregulated the phosphorylation levels of PI3K, AKT, ERK, and downregulated the phosphorylation level of NF-κB (*P* < 0.05). While administration of LY294002 reduced cell viability, increased cell apoptosis, and aggravated inflammatory response (*P* < 0.05).

**Conclusion:** Our results suggested that the protective effect of osmotin on the simulated OGD/R injured H9c2 cells might be associated with AdipoR1/PI3K/AKT signaling pathway.

## Introduction

Acute myocardial infarction (AMI) is a common and lethal heart disease which threatens people's life worldwide (Santos-Gallego et al., 2014). Timely reperfusion is critical for the salvage of ischemic myocardium. Although reperfusion was well acknowledged to protect the ischemic myocardium against inevitable death, it also has side effect which was called ischemia/reperfusion (I/R) injury (Heusch, 2013). Myocardial I/R injury is a very complex pathophysiological process that can lead to serious acute and chronic myocardial damage. In addition to cell necrosis, cell apoptosis is another important cause which induce cell death during myocardial I/R. Moreover, it has been well demonstrated that a cascade of acutely initiated local inflammatory responses were one of the most important pathological processes during I/R (Frangogiannis et al., 2002; Kawaguchi et al., 2011). Therefore, reduction of cell apoptosis and inflammatory responses might be of great importance.

Osmotin is a 24 kDa protein which belongs to pathogenesis-related protein of group 5 (PR-5) (Min et al., 2003). It plays a critical role in plants via defensing against pathogens (Yun et al., 1998). Osmotin is a homolog of mammalian adiponectin, adiponectin and osmotin do not share sequence similarity at the amino acid level. But interestingly, osmotin is structurally and functionally similar to mammalian adiponectin (Anil et al., 2015), and adiponectin has been widely reported to exert cardioprotective effects during myocardial I/R (Ouchi et al., 2006; Gonon et al., 2008). Dietary osmotin is considered as naturally occurring adiponectin receptors (AdipoRs) agonist (Yamauchi and Kadowaki, 2008). An increasing number of studies have suggested that osmotin is involved in pathological processes, such as cell apoptosis and inflammatory response (Narasimhan et al., 2005; Shah et al., 2014; Ullah et al., 2014; Badshah et al., 2016). Osmotin has been reported to exert protective effects on many brain disorders, including amyloid beta (Aβ)-induced memory impairment, tau phosphorylation, synaptic dysfunction, and neurodegeneration (Naseer et al., 2014; Shah et al., 2014; Ali et al., 2015). Recently, a study found that osmotin might exert its beneficial effects on Alzheimer's disease (AD) by regulation of AdipoR1/AMP-activated protein kinase (AMPK) pathways. Moreover, it has been reported that adiponectin/AdipoRs protected the heart from I/R injury through AMPK pathway (Shibata et al., 2005; Gonon et al., 2008). In consideration of the regulation effects, we speculated that osmotin might have protective effects on myocardial I/R by modulation of AdipoRs.

Phosphoinositide 3-kinase (PI3K) and the downstream target serine/threonine kinase AKT constitute a powerful survival signaling pathway, which is involved in regulating cell survival and growth, inflammatory responses, and apoptosis (Cantley, 2002). Increasing researches suggested that activation of PI3K/AKT-dependent signaling could inhibit cardiac myocyte apoptosis and protect myocardial I/R injury (Fujio et al., 2000; Ha et al., 2008). It has been reported that osmotin protected brain against diseases by activation of PI3K/AKT (Ali et al., 2015; Shah et al., 2016). However, it is unclear whether the effects of osmotin on myocardial I/R injury is related with PI3K/AKT pathway. Therefore, in the present study, we aimed to investigate the effect of osmotin on myocardial I/R, as well as underlying mechanism and potential signaling pathway. Our data might provide a novel potential of osmotin as a therapeutic strategy for myocardial I/R injury.

## Materials and methods

### Cell culture

H9c2 cell line derived from rat primary cardiomyocytes was purchased from the American Type Culture Collection (ATCC; Manassas, VA, USA). The cells were cultured in Dulbecco's modified Eagle's medium (DMEM; Sigma-Aldrich, St Louis, USA) supplemented with 10% (v/v) fetal bovine serum (FBS; GIBCO, Grand Island, NY, USA), 100 U/mL penicillin (GIBCO), 100 μg/mL streptomycin (GIBCO) and 2 mM glutamine (GIBCO). The cells were maintained in a humidified incubator with 95% air and 5% CO_2_ at 37°C.

### Simulation of myocardial I/R injury model and treatment

In order to mimic an *in vitro* model of myocardial I/R injury, H9c2 cells were subjected with the oxygen and glucose deprivation followed by reperfusion (OGD/R). OGD was initiated as previously described (Wu et al., 2013). Briefly, cells were seeded into 35 mm plates at a density of 3 × 10^5^ cells/well and cultured for 24 h. Then, the cell culture medium was replaced with glucose-free DMEM, and the cells were maintained in an anaerobic chamber in the oxygen-free incubator (95% N_2_ and 5% CO_2_) at 37°C for 4 h. Subsequently, the glucose content in culture medium was adjusted to 4.5 mg/mL, and the cells were incubated under 95% air and 5% CO_2_ at 37°C for another 24 h.

Osmotin was dissolved in water to a concentration of 0.1–1.0 mg/mL. For extended storage, it is dissolved in a buffer containing 0.1% BSA (Sigma-Aldrich) and store in working aliquots at −20°C to −80°C to further dilute as manufacturer's instructions recommend. The cells were exposed to vehicle (DMSO), osmotin (0.05–0.3 μM; Sigma-Aldrich) and/or LY294002 (20 μM; Sigma-Aldrich) (Ishii et al., 2015) under OGD/R procedures, respectively. Cells in normal DMEM medium and been cultured at 37°C in a 95% air and 5% CO_2_ atmosphere were used as control.

**Figure 1:**
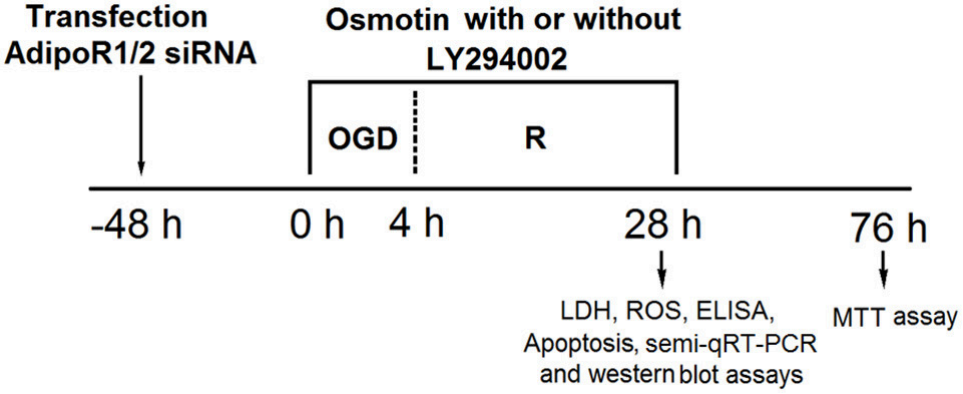
The time axis of OGD/R exposure and osmotin administration with or without LY294002 treatment. OGD/R, oxygen and glucose deprivation/reperfusion; AdipoR, adiponectin receptor; siRNA, small interfering RNA; semi-qRT-PCR, Semi-quantitative real-time reverse transcriptase polymerase chain reaction; LDH, lactate dehydrogenase; ROS, reactive oxidative stress; MTT, 3-(4, 5-dimethylthiazol-yl)-2, 5-diphenyl-2-H-tetrazolium bromide.

The time axis of OGD/R exposure and osmotin administration with or without LY294002 treatment was provided in Figure 1.

### Cell transfection

Small interfering RNAs (siRNAs) with sequences specially targeting AdipoR1 or AdipoR2 were designed and synthesized by GenePharma (Shanghai, China). The sequences of the siRNAs were provided in Supplementary Table 1. They were constructed and packaged by chitosan nanoparticle to been transfected into H9c2 cells. For stable transfection, the cells at a density of 5 × 10^5^ cells/per well were seeded on 6-well plates and then been transiently transfected with 50 nM specific siRNAs according to the manufacturer's instruction. The transfection was performed by using Lipofectamine 2000 (Invitrogen, USA). After 48 h of transfection, the cell suspension was collected for further analyses. Untreated cells were regarded as control.

### Cell viability assay

The cell viability was analyzed by a 3-(4, 5-dimethylthiazol-yl)-2, 5-diphenyl-2-H-tetrazolium bromide (MTT) colorimetric assay according to a standardized method (Inada et al., 2011). Briefly, the cells were seeded on 96-well plates for adherence. After corresponding administration and another 48 h of incubation without any treatment in normal conditions, the cells were added with 5 mg/mL MTT (20 μL; Sigma-Aldrich) and incubated at 37°C for 4 h. Then, the cells were added with 100 μL dimethylsulfoxide (DMSO; Sigma-Aldrich) to dissolve the formazan crystals. The absorbance at 590 nm was read by using microplate reader (Bio-Rad Benchmark, Hercules, CA, USA).

### Lactate dehydrogenase (LDH) release activity assay

Cell damage was also assessed by measurement of LDH release activity after corresponding administration by using a LDH-Cytotoxicity Detection Kit (Roche, Mannheim, Germany) according to the instructions. The absorbance value of 492 nm was measured by a spectrometer (Lab Tech, Boston, Massachusetts, USA). Cells of control group were treated with 2% Triton-100 (GIBCO, USA) and the detection result was regarded as the total LDH activity. The related LDH release activity was assessed according to the following equation: LDH release = (LDH activity in the medium/total LDH activity) × 100%.

### Apoptosis quantification

The cell apoptosis was determined by using the FITC Annexin V Apoptosis Detection Kit (BD Biosciences, San Diego, CA) and quantified by flow cytometry. Briefly, after corresponding administration, the cells were transferred into culture tube, and been added with a solution containing 5 μL Annexin V-FITC and 10 μL PI. The cells were incubated at room temperature in the dark for 15 min, followed by addition of 300 μL binding buffer supplemented in the detection kit. The cells were then immediately analyzed by flow cytometry. Flow cytometric analysis was performed with a FACScan flow cytometer (Becton Dickinson, San Jose, CA, USA), using the Cell Quest software (BD, San Diego, CA, USA).

### Reactive oxidative stress (ROS) detection

ROS generation was measured by using 2sing-Dichlorofluorescin diacetate (DCFDA; Sigma-Aldrich) reagent. Cells were seeded in 96-well plants, and were treated with corresponding administration. Then, 600 mM DCFDA solution dissolved in PBS was added into each well, and the plants were allowed for culturing at 37°C for 30 min. The plates were then read in ApoTox-Glo (Promega, Madison, WI, USA) at 488/530 nm.

### Semi-quantitative real-time reverse transcriptase polymerase chain reaction (semi-qRT-PCR)

Total RNA was extracted from the cells with TRIzol (Invitrogen, USA) according to the manufacturer's instructions. Template cDNA was prepared and then was amplified with qPCR primers designed and synthesized by GenePharma (Shanghai, China). Reverse transcription was performed on an ABI 7500 Fast Dx real-time PCR system (Thermo Fisher Scientific, Inc.) according to the following program: reverse transcription at 50°C for 30 min, initial denaturation at 94°C for 2 min, and then 45 cycles of 95°C for 15 s, 55°C for 1 min, and 72°C for 5 s. RT-PCR was carried out with a LightCycler 2.0 (Roche) using the SYBR® Advantage® qPCR Premix Kit (Clontech, Mountain View, CA). PCR primer sequences of *AdipoR1, AdipoR2* and *GAPDH*, used as an internal control, were shown in Supplementary Table 2.

### Western blot

After corresponding treatments, the cells suspension was harvested, and been centrifuged, then been lysed in a RIPA lysis buffer with protease and phosphatase inhibitor cocktails. The protein samples were quantified by the Bradford assay (Thermo, Hercules, CA). Equal amounts of samples were separated by 10–12% sodium dodecyl sulfate (SDS)-polyacrylamide gel electrophoresis (PAGE) gels, and then were transferred onto nitrocellulose membranes. Thereafter, the membranes were incubated with 5% skim milk in Tris Buffered Saline with Tween (TBST) for 1 h and probed with the following primary antibodies overnight at 4°C: anti-AdipoR1 antibody (ab126611; Abcam, Cambridge, UK), anti-AdipoR2 antibody (ab77612; Abcam), anti-Bax antibody (ab32503; Abcam), anti-B-cell lymphoma (Bcl)-2 antibody (ab32124; Abcam), anti-pro-caspase-3 antibody (ab32150; Abcam), anti-cleaved caspase-3 antibody (ab2302; Abcam), anti-IL-4 (ab89974; Abcam), anti-IL-10 (ab9969; Abcam), anti-IL-13 (ab106732; Abcam), anti-ERK1/2 (ab17942; Abcam), anti-interleukin (IL)-1β antibody (MABF18; Sigma-Aldrich), anti-IL-6 antibody (SAB3500310; Sigma-Aldrich), anti-IL-8 antibody (SAB4504243; Sigma-Aldrich), anti-tumor necrosis factor (TNF)-α antibody (T0938; Sigma-Aldrich), anti-nuclear factor (NF)-κB antibody (SAB4502609; Sigma-Aldrich), anti-PI3K antibody (#4249; Cell signaling Technology), anti-p-PI3K antibody (#4228; Cell signaling Technology), anti-AKT antibody (#4691; Cell signaling Technology), anti-p-Akt antibody (#4060; Cell signaling Technology), anti-p-ERK1/2 (#9101; Cell signaling Technology), anti-p-NF-κB (orb106093; Biorbyt, Cambridge, UK). GAPDH was used as an internal control. Membranes were then washed three times with TBST buffer and incubated with the appropriate horseradish peroxidase-conjugated secondary antibodies (Cell signaling Technology) for 2 h at room temperature. Images were acquired with WEST-ZOL-plus Western Blot Detection System (Intron Biotechnology, Inc., Korea) using enhanced chemiluminescent (ECL) reagents according to the manufacturer's instructions and analyzed by ImageJ 1.49 (National Institute of Health, Bethesda, MD).

### Enzyme immunoassay for cytokines quantitative

The concentrations of the inflammatory cytokines, including TNF-α, IL-1β, IL-8, and IL-6, were determined by ELISA kit (Thermo Scientific, Waltham, MA, USA). Optical density was measured at 450 nm. The data, expressed as pictogram per milliliter, were calculated on the basis of linear calibration curves generated with standard solutions.

### Statistical analysis

All samples were run in triplicate and experiments were repeated at least five times. The data are presented as the mean ± SEM. Statistical analyses were performed using SPSS version 19.0 program (SPSS Inc., Chicago, IL, USA). The *P*-values were calculated using one-way ANOVA with Duncan procedure. *P*-value of <0.05 was considered statistically significant.

## Results

### Effects of osmotin and silencing of AdipoRS on cell viability

To start with, various dose of osmotin (0–0.3 μM) were used to treat H9c2 cells, and the changes in cell viability were monitored. As data given in Supplementary Figure 1A, no significant difference in cell viability was observed between control (0 μM) and low-dose of osmotin (0.05, 0.10, 0.15, and 0.20 μM) groups (*P* > 0.05). Cells treated with high-dose of osmotin (0.25 and 0.3 μM) resulted in significant decreases in cell viability (*P* < 0.05). The effects of various dose of osmotin on cell viability were also detected when cells were subjected with OGD/R-stimulating conditions. Results in Supplementary Figure 1B indicated that, osmotin could rescue cell from OGD/R-induced cell viability impairment, as cell viability was significantly increased by addition of osmotin (*P* < 0.05). An interesting feature of the osmotin on cell viability is that the highest growth acceleration is achieved at addition level of 0.2 μM rather than other levels. Thus, the osmotin is fixed at 0.2 μM and is selected for the later study.

**Figure 2:**
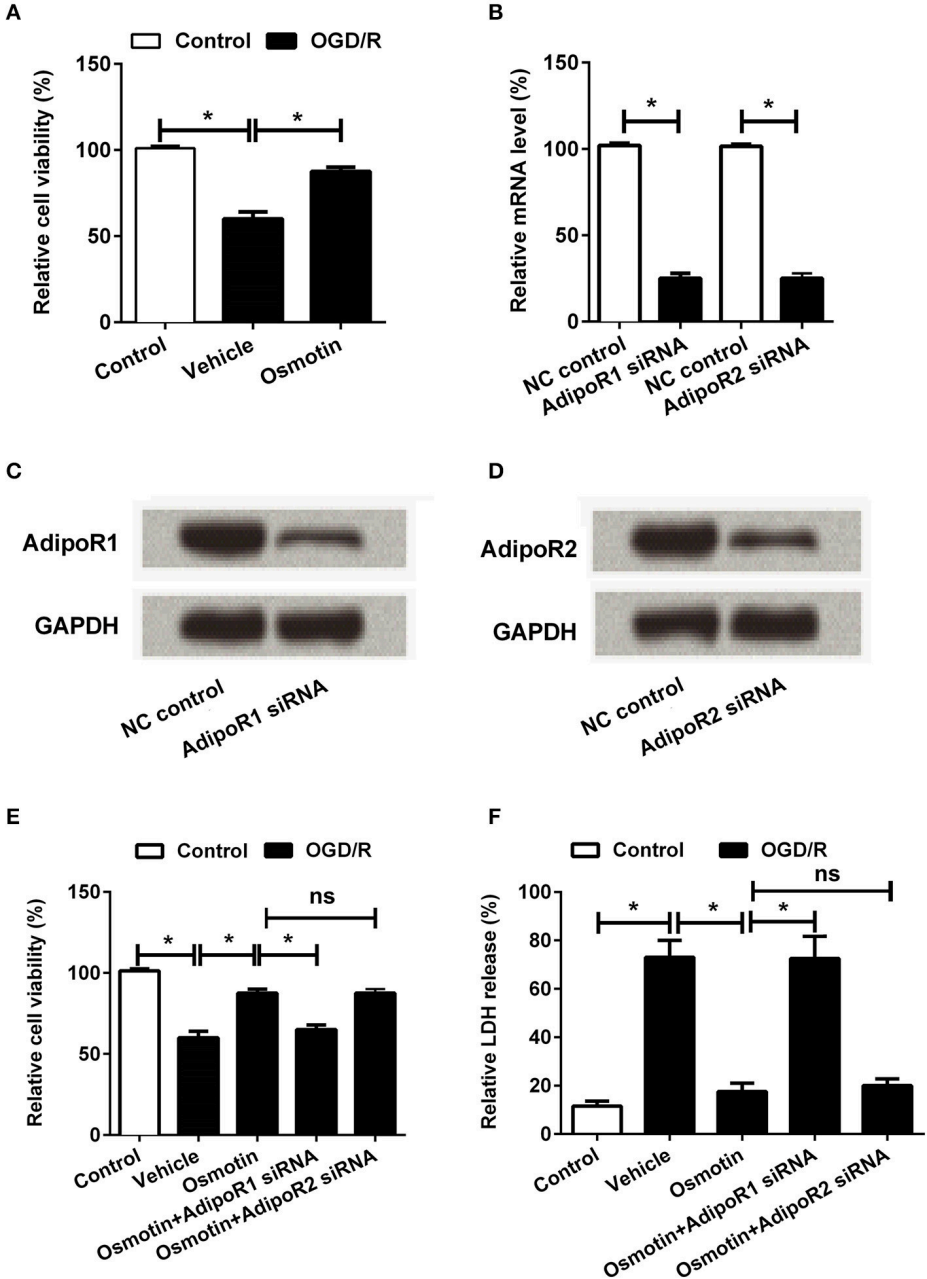
Effects of osmotin combine with AdipoRs silence on H9c2 cell viability. H9c2 cells were pre-treated with OGD/R, and then been administrated with osmotin and/or transfection with specifically siRNAs for AdipoR1 or AdipoR2. **(A)** Effects of osmotin on the cell viability (*n* = 5); **(B–D)**. Transfection efficiency of siRNAs for AdipoR1 and AdipoR2 were measured by semi-qRT-PCR (*n* = 5), and western blot assay. GAPDH acted as internal control; **(E)** Effects of osmotin and/or combine with AdipoR1 or AdipoR2 siRNAs transfection on cell viability (*n* = 5). **(F)** Relative LDH release of H9c2 cells after corresponding administrations (*n* = 5). ^*^P < 0.05 compare to corresponding control; ns, no significant compared to the osmotin group. OGD/R, oxygen and glucose deprivation/reperfusion; AdipoR, adiponectin receptor; NC, negative control; siRNA, small interfering RNA; semi-qRT-PCR, semi-quantitative Real-time reverse transcriptase polymerase chain reaction; LDH, lactate dehydrogenase.

To investigate the functional role of osmotin in OGD/R-induced injury of H9c2 cells, the cells were pre-treated with 0.2 μM osmotin and then cell viability was measured under OGD/R condition by MTT. Cells without treatment were regarded as control group. As shown in Figure 2A, the MTT results showed that the cell viability was significantly decreased by OGD/R induction compared to the control group without treatment (*P* < 0.05), while the cell viability was statistically increased after osmotin treatment compare with vehicle under OGD/R induction (*P* < 0.05). It suggested that osmotin might protect H9c2 cells viability to against OGD/R-induced injury.

To further investigate the underlying mechanism of osmotin protected H9c2 cell against injury, downstream receptors of osmotin AdipoR1 and AdipoR2 were knocked down by specific siRNAs transfection. The transfection efficiency was confirmed by semi-qRT-PCR and western blot, respectively. Both the mRNA (Figure 2B) and protein (Figures 2C,D) levels of AdipoR1 and AdipoR2 were markedly reduced after specific siRNAs transfection compared to negative control group (*P* < 0.05 for mRNA expression level analysis), suggesting the high transfection efficiency of siRNAs. Subsequently, the roles of AdipoR1 and AdipoR2 in osmotin effect on H9c2 cells were explored by cell viability assay and LDH release activity assay. The results showed that knockdown of AdipoR1 decreased cell viability compared with osmotin treated H9c2 cells (*P* < 0.05), while the AdipoR2 siRNA administrated group showed no significant difference compare with osmotin treated group (*P* > 0.05; Figure 2E). The results of LDH release activity assay showed that osmotin treatment decreased LDH release compared with vehicle as control under OGD/R induction (*P* < 0.05), while AdipoR1 siRNA transfection increased it compare with osmotin alone treatment (P < 0.05). AdipoR2 siRNA transfection showed no significantly effect on LDH release activity compared with osmotin alone treatment (*P* > 0.05; Figure 2F). These results suggested that the protective effect of osmotin might via modulation of AdipoR1, but not AdipoR2.

### Effects of osmotin and AdipoRs silence on cell apoptosis

**Figure 3:**
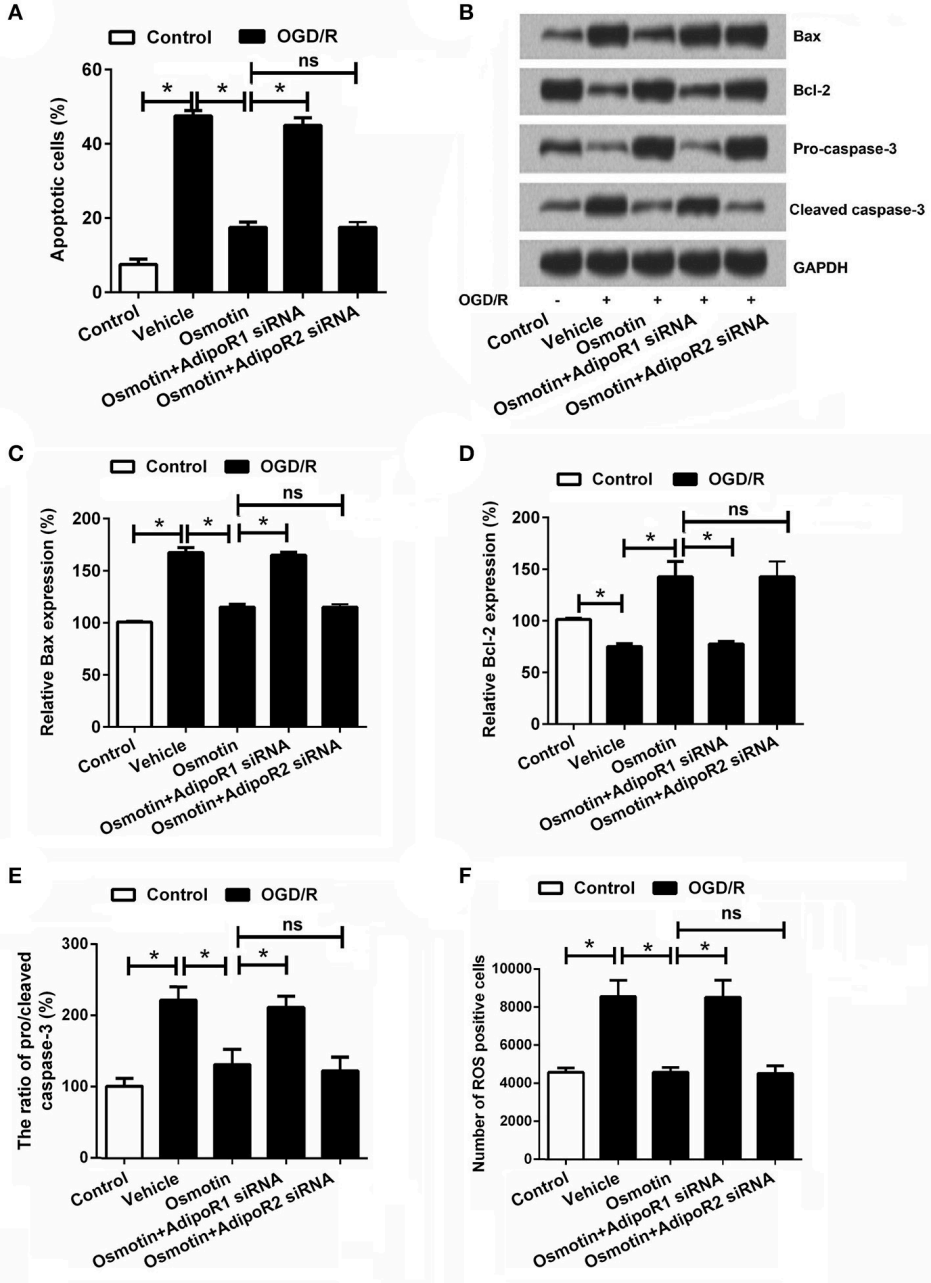
Effects of osmotin and silencing of AdipoRs on apoptosis of H9c2 cells. H9c2 cells were pre-treated with OGD/R, and then been administrated with osmotin and/or transfection with specifically siRNAs for AdipoR1 or AdipoR2. **(A)** Effects of osmotin and silencing of AdipoRs on the percentages of apoptotic cells (*n* = 5); **(B)** The expressions of apoptosis-related proteins were measured by Wearer blot. **(C–E)** Relative protein expression levels of apoptotic related proteins (*n* = 5). **(F)** Effects of osmotin and silencing of AdipoRs on the number of ROS positive cells (*n* = 5). ^*^P < 0.05 compare to corresponding control; ns, no significant compared to the osmotin group. OGD/R, oxygen and glucose deprivation/reperfusion; AdipoR, adiponectin receptor; Bcl, B-cell lymphoma; siRNA, small interfering RNA; ROS, reactive oxidative stress; ns, non-significant.

It has been well demonstrated that I/R injury results in cell apoptosis; therefore, we investigated the roles of osmotin in this process. The cell apoptosis was analyzed by flow cytometry. As shown in Figure 3A, we found that OGD/R significantly increased the percentage of apoptotic cell compared to the control group without treatment (*P* < 0.05), while osmotin dramatically decreased the percentages of apoptotic cell compared to the vehicle group under OGD/R condition (*P* < 0.05). In addition, the results showed that AdipoR1 siRNA transfection increased cell apoptosis compare with the osmotin group (*P* < 0.05); however, AdipoR2 siRNA transfection had no significantly influence on cell apoptosis compare with osmotin (*P* > 0.05). We then explored the mechanism about apoptosis by determining the protein expressions of apoptosis related proteins (Bax, Bcl-2, cleaved caspase-3, and pro-caspase-3). As shown in Figures 3B–E, western blot analysis results showed that osmotin significantly decreased expression of pro-apoptotic protein Bax and cleaved-caspase-3, while increased anti-apoptotic protein Bcl-2 and pro-caspase-3 expressions compared with those in vehicle groups under OGD/R condition (*P* < 0.05). AdipoR1 siRNA transfection increased these factors expressions compare with osmotin (*P* < 0.05), while AdipoR2 siRNA transfection group showed no significantly compared with osmotin alone (*P* > 0.05). These data suggested that osmotin protected cells against OGD/R-induced apoptosis via regulation of apoptosis related proteins expressions and this effect might be related with AdipoR1.

Considering that overproduction of ROS can induce apoptosis through both extrinsic and intrinsic pathways (Zhang et al., 2017), we further detected ROS generation of the H9c2 cells following by osmotin treatment and OGD/R exposure. Results in Figure 3F showed that, the number of ROS positive cell was significantly increased in response to OGD/R induction (*P* < 0.05), and was significantly reduced by addition to osmotin (*P* < 0.05). As expected, osmotin did not decrease the number of ROS positive cell under OGD/R-stimulating conditions when AdipoR1 was knocked down (*P* < 0.05), rather than AdipoR2 knocked down (*P* > 0.05).

### Effects of osmotin and AdipoRs silence on the expression of inflammatory factors

**Figure 4:**
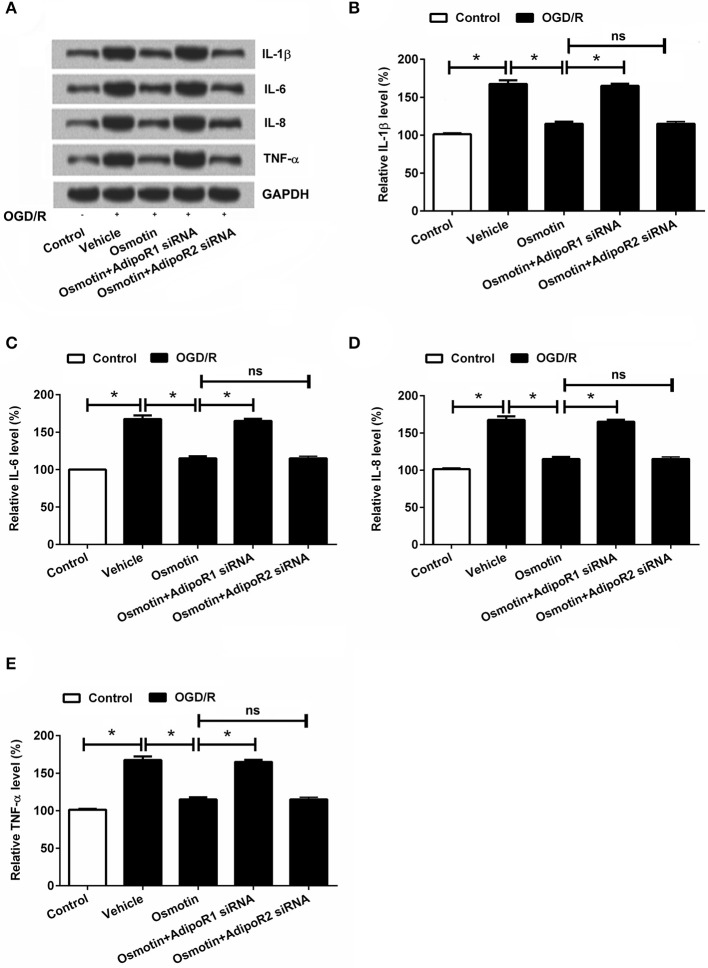
Effects of osmotin and silencing of AdipoRs on expressions of proinflammatory factors in H9c2 cells. H9c2 cells were pre-treated with OGD/R, and then been administrated with osmotin and/or transfection with specifically siRNAs for AdipoR1 or AdipoR2. **(A)** Expressions of proinflammatory factors including IL-1β, IL-6, IL-8, and TNF-αmeasured by western blot. **(B–E)**, Relative protein expression levels of proinflammatory factors IL-1β, IL-6, IL-8, and TNF-α(*n* = 5). ^*^P < 0.05 compare to corresponding control; ns, no significant compared to the osmotin group. OGD/R, oxygen and glucose deprivation/reperfusion; AdipoR, adiponectin receptor; IL, interleukin; TNF, tumor necrosis factor; NF-κB, nuclear factor-kappa B; siRNA, small interfering RNA; ns, non-significant.

**Figure 5:**
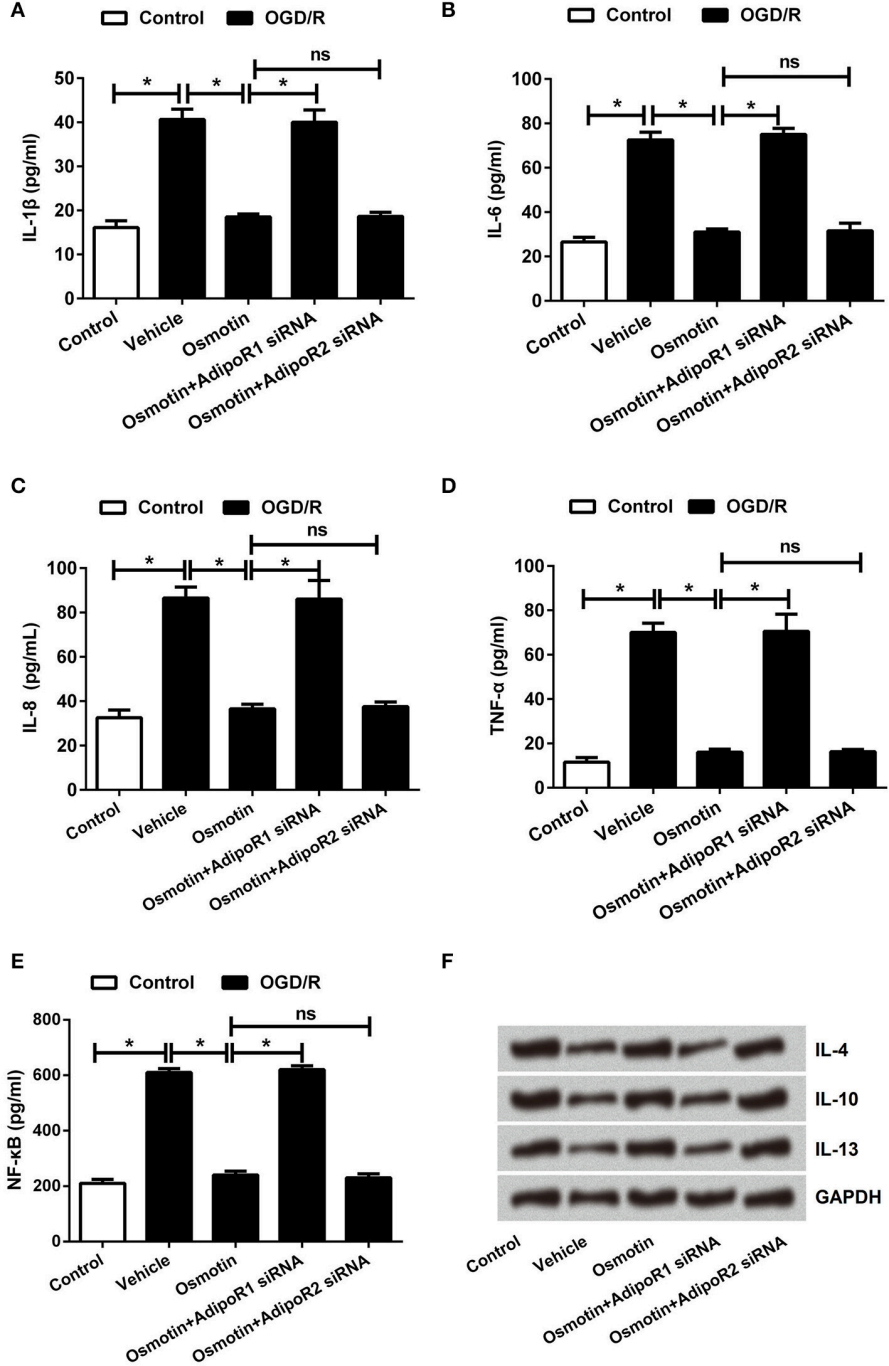
Effects of osmotin and silencing of AdipoRs on inflammatory factors in H9c2 cells. H9c2 cells were pre-treated with OGD/R, and then been administrated with osmotin and/or transfection with specifically siRNAs for AdipoR1 or AdipoR2. **(A–D)**. The relative contents of proinflammatory factors IL-1β, IL-6, IL-8, and TNF-α were measured by ELISA assay (*n* = 5). **(E)** Protein expressions of anti-inflammatory factors IL-4, IL-10 and IL-13 were measured by western blot. ^*^P < 0.05 compare to corresponding control; ns, no significant compared to the osmotin group. OGD/R, oxygen and glucose deprivation/reperfusion; AdipoR, adiponectin receptor; IL, interleukin; TNF, tumor necrosis factor; siRNA, small interfering RNA; ns, non-significant.

Subsequently, the effects of osmotin on proinflammatory factor expressions were analyzed. The mRNA and protein expressions of IL-1β, IL-6, IL-8, and TNF-αin H9c2 cells were determined. The results in Figures 4A–E showed that the expression levels of IL-1β, IL-6, IL-8, and TNF-α were all significantly increased by OGD/R compared to the control group without treatment (*P* < 0.05), these factors expressions were statistically reduced by osmotin administration compared to vehicle group (*P* < 0.05). Besides, we observed that osmotin + AdipoR1 siRNA dramatically increased the levels of IL-1β, IL-6, IL-8, and TNF-α compared to the group only administration with osmotin (*P* < 0.05), while osmotin + AdipoR2 silence had no effects on the expression of proinflammatory factors. To further validate the effect of osmotin on these factor expressions, their contents in cell were also assessed by ELISA. The results in Figures 5A–D showed that contents of these factors were increased in OGD/R administration group compare with control group without treatment (*P* < 0.05). Among OGD/R groups, osmotin administration decreased these factors contents compared to the vehicle group (*P* < 0.05). AdipoR1 siRNA transfected groups showed decreased contents of these factors compared with osmotin alone treatment (*P* < 0.05), while AdipoR2 siRNA transfected group showed no significantly (*P* > 0.05) compare with osmotion alone. Also, the expressions of anti-inflammatory factors, including IL-4, IL-10 and IL-13 were also detected. We found that IL-4, IL-10 and IL-13 were all downregulated by OGD/R stimulation, and were upregulated by addition to osmotin (Figure 5E). As expected, AdipoR1 siRNA transfected groups showed downregulated expressions of these factors compared with osmotin alone treatment, while AdipoR2 siRNA transfected group showed no impact compare with osmotion alone. These results suggested that osmotin might inhibit OGD/R-induced inflammatory response via regulating AdipoR1 expression.

### Effects of osmotin and silencing of AdipoRs on PI3K/AKT, NF-κB, and ERK pathways

**Figure 6:**
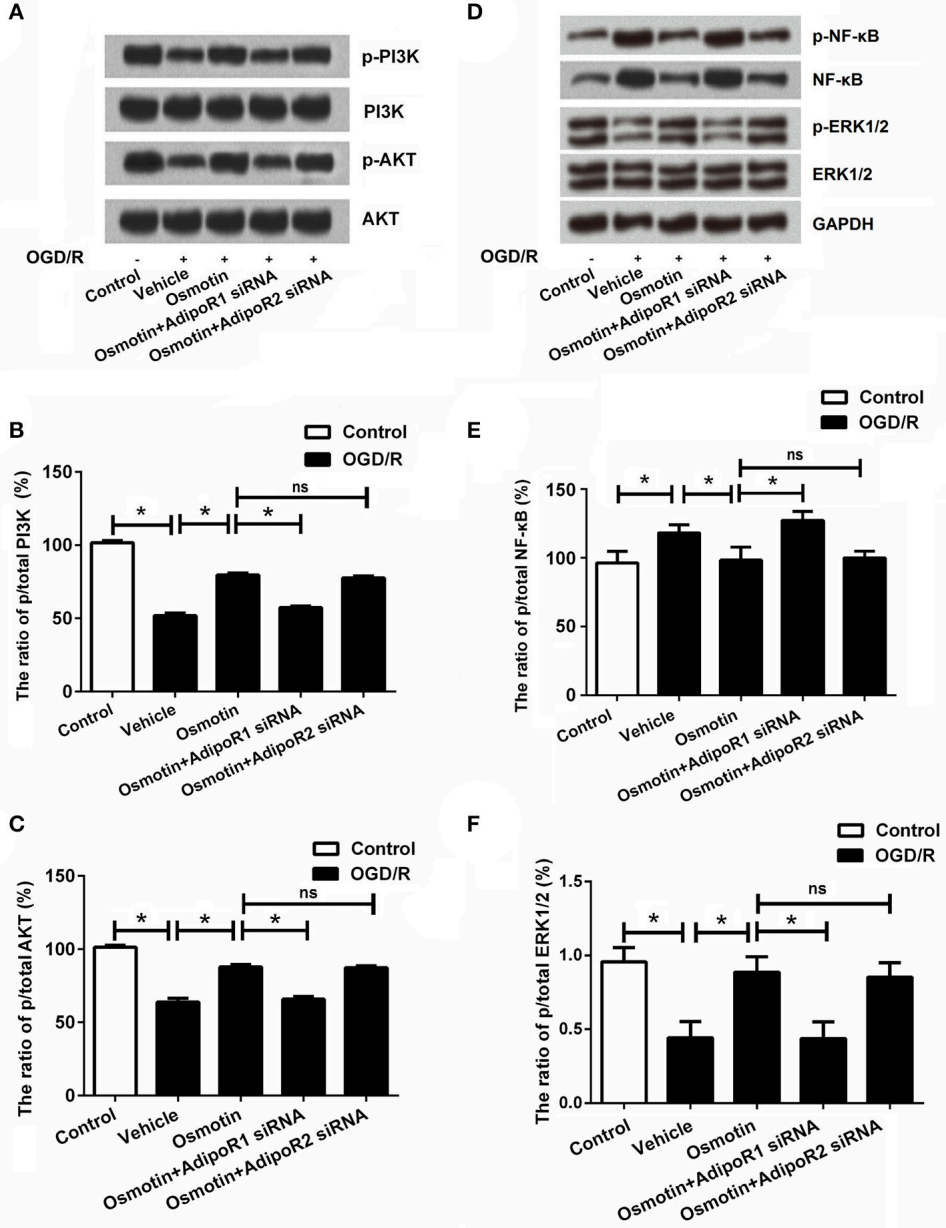
Effects of osmotin and silencing of AdipoRs on the expressions of PI3K, AKT, NF-κB, and ERK in H9c2 cells. H9c2 cells were pre-treated with OGD/R, and then been administrated with osmotin and/or transfection with specifically siRNAs for AdipoR1 or AdipoR2. **(A)** Expressions of p/t-PI3K and p/t-AKT in H9c2 cells measured by western blot after corresponding administrations. **(B)** The ratio of p/t PI3K (*n* = 5). **(C)** The ratio of p/t AKT (*n* = 5). **(D)** Expressions of p/t-NF-κB and p/t-ERK1/2 in H9c2 cells measured by western blot after corresponding administrations. **(E)** The ratio of p/t NF-κB. **(F)** The ratio of p/t ERK1/2 ^*^P < 0.05 compare to corresponding control; ns, no significant compared to the osmotin group. OGD/R, oxygen and glucose deprivation/reperfusion; AdipoR, adiponectin receptor; PI3K, phosphatidylinositol 3-kinase; NF-κB, nuclear factor kappa B; siRNA, small interfering RNA; ns, non-significant.

We analyzed the effect of osmotin on activating of PI3K/AKT, NF-κB and ERK signaling by measuring expression levels of phosphorylated (p)-PI3K, p-AKT, p-NF-κB, and p-ERK1/2. The western blot analysis results in Figures 6A–F showed that expressions of p-PI3K, p-AKT, and p-ERK1/2 were significantly lower, while p-NF-κB was significantly higher in H9c2 cells with OGD/R-induced injury compared with that in untreated cells (*P* < 0.05). These data suggested OGD/R inactivated PI3K/AKT and ERK pathways, while activated NF-κB pathway. Of contrast, the expression levels of p-PI3K, p-AKT, and p-ERK1/2 were statistically increased, and the expression level p-NF-κB was significantly decreased by osmotin compared to the vehicle group (*P* < 0.05), suggesting osmotin promoted the activation of PI3K/AKT and ERK pathways and inhibited the activation of NF-κB pathway in H9c2 cells. Moreover, osmotin did not activated PI3K/AKT and ERK pathways, and did not inactivated NF-κB pathway when AdipoR1 was knocked down, rather than when AdipoR2 was knocked down. These results suggested that osmotin might be related with activation of PI3K/AKT, ERK, and NF-κB signaling pathways via AdipoR1.

### Involvement of PI3K/AKT pathway in the protective effect of osmotin on OGD/R-induced injury of H9c2 cells

**Figure 7:**
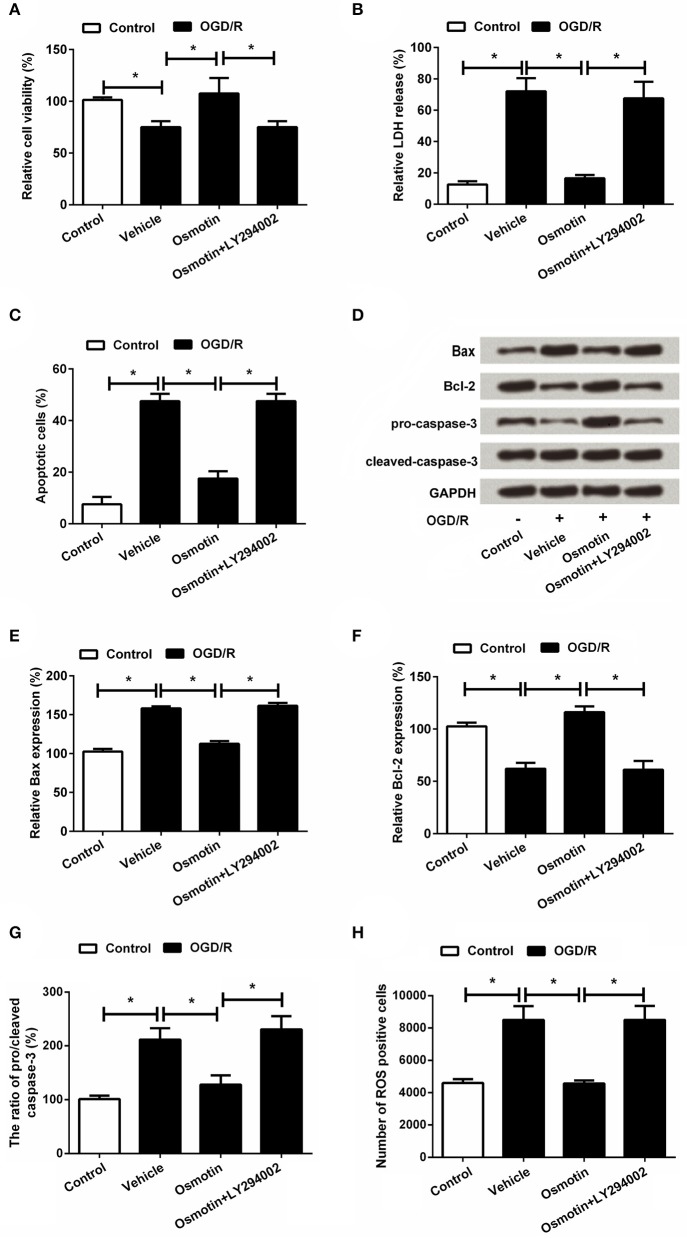
Effects of osmotin and administration of LY294002 on H9c2 cell viability and apoptosis. H9c2 cells were pre-treated with OGD/R, and then been administrated with osmotin and/or LY294002 as inhibitor of PI3K. **(A)** Effects of osmotin and/or LY294002 administration on cell viability (*n* = 5); **(B)** Relative LDH release of administrated H9c2 cells (*n* = 5). **(C)** Effects of osmotin and administration of LY294002 on cell apoptosis (*n* = 5). **(D)** Expressions of apoptotic related factors measured by western bolt. *GAPDH* acted as internal control. **(E–G)** Relative expression levels of apoptotic related factors (*n* = 5). **(H)** Effects of osmotin and/or LY294002 administration on the number of ROS positive cells (*n* = 5). ^*^P < 0.05 compare to corresponding control. OGD/R, oxygen and glucose deprivation/reperfusion; LY294002, inhibitor of phosphatidylinositol 3-kinase (PI3K); LDH, lactate dehydrogenase; ROS, reactive oxidative stress; LY294002, inhibitor of phosphatidylinositol 3-kinase (PI3K).

Based on the above results, we speculated that the protective effect of osmotin on OGD/R-induced injury in H9C2 cells might be at least in part via AdipoR1/PI3K/AKT pathway. Therefore, H9c2 cells were administrated by the inhibitor of PI3K (LY294002) and then the effects of osmotin on cell viability and apoptosis were assessed. As shown in Figure 7, results showed that osmotin + LY294002 treatment markedly decreased H9c2 cells viability (Figure 7A), and also obviously increased LDH release (Figure 7B) compared with osmotin alone administration under OGD/R condition (*P* < 0.05). Also combination of osmotin + LY294002 increased the percentages of apoptotic cells (Figure 7C) compared to osmotin administration alone (*P* < 0.05), as well as the expressions of apoptotic related factors (*P* < 0.05; Figures 7D–G). Moreover, the number of ROS positive cells was significantly increased in response to a combination of osmotin + LY294002 (*P* < 0.05; Figure 7H).

**Figure 8:**
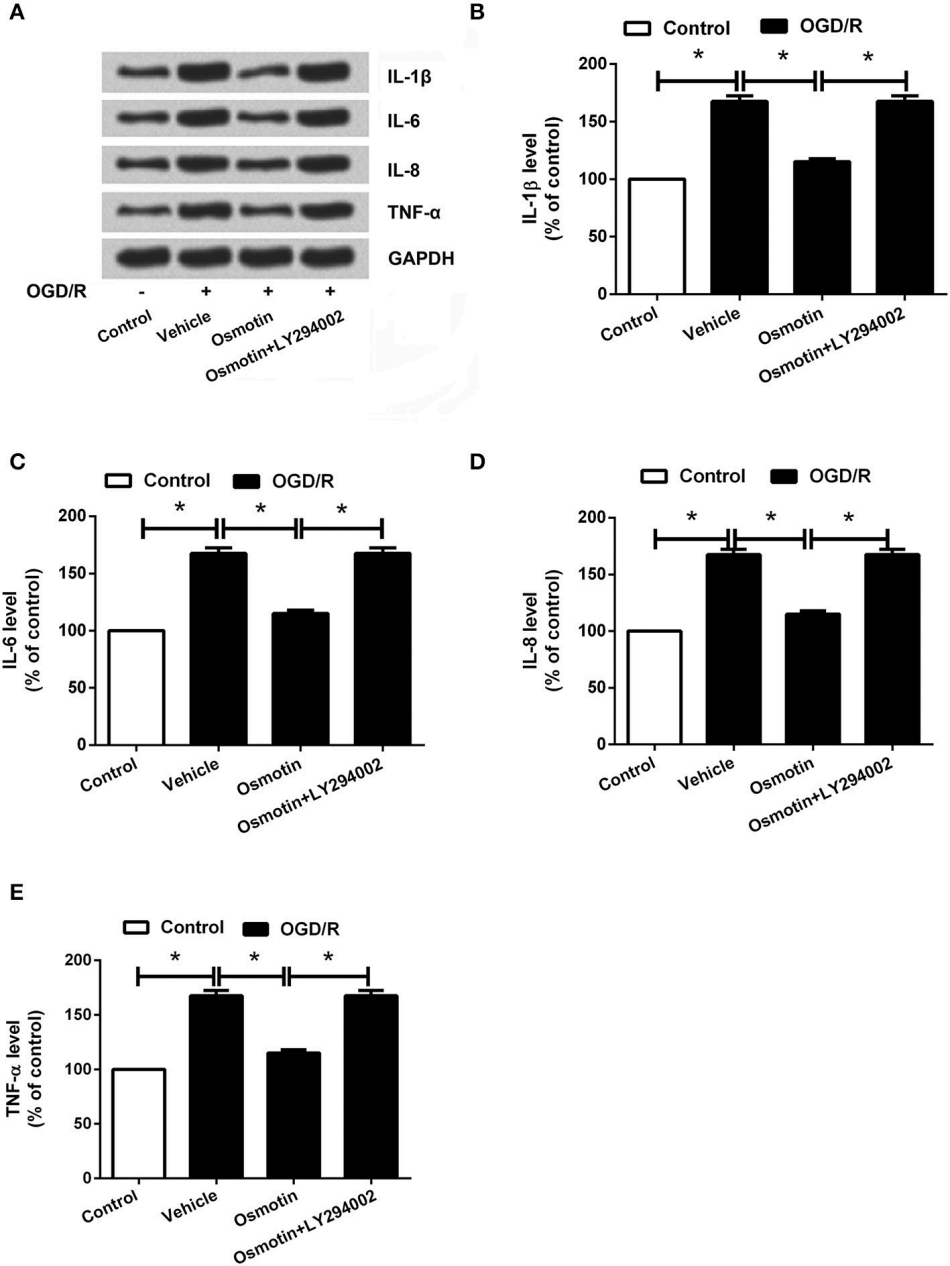
Effects of osmotin and administration of LY294002 on the proinflammatory factors expressions in H9c2 cells. H9c2 cells were pre-treated with OGD/R, and then been administrated with osmotin and/or LY294002 as inhibitor of PI3K. **(A)** Expressions of IL-1β, IL-6, IL-8, and TNF-α in H9c2 cells after administration were measured by western blot. **(B–E)** Relative expression levels of IL-1β, IL-6, IL-8, and TNF-α(*n* = 5). ^*^P < 0.05 compare to corresponding control. OGD/R, oxygen and glucose deprivation/reperfusion; LY294002, inhibitor of phosphatidylinositol 3-kinase (PI3K); IL, interleukin; TNF, tumor necrosis factor.

**Figure 9:**
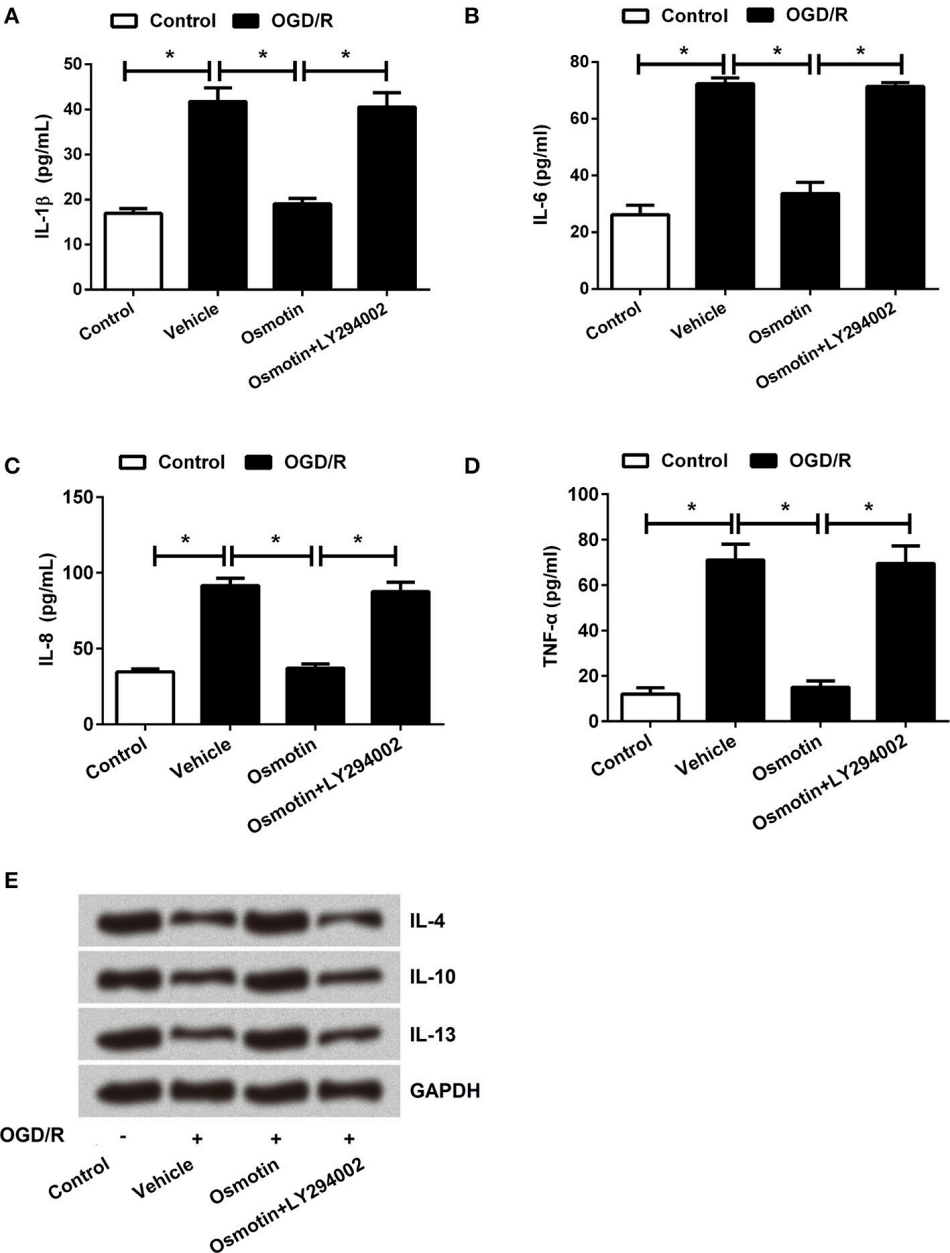
Effects of osmotin and administration of LY294002 on the inflammatory factors contents in H9c2 cells. H9c2 cells were pre-treated with OGD/R, and then been administrated with osmotin and/or LY294002 as inhibitor of PI3K. **(A–D)** Results of ELISA assay about the contents of proinflammatory factors IL-1β, IL-6, IL-8, and TNF-α(*n* = 5). **(E)** Protein expressions of anti-inflammatory factors IL-4, IL-10 and IL-13 were detected by western blot ^*^P < 0.05 compare to corresponding control. OGD/R, oxygen and glucose deprivation/reperfusion; AdipoR, adiponectin receptor; LY294002, inhibitor of phosphatidylinositol 3-kinase (PI3K); IL, interleukin; TNF, tumor necrosis factor.

Furthermore, we found that osmotin + LY294002 administration increased the proinflammatory factors expressions and the contents of these factors in H9c2 cells after been treated compared with osmotin alone administration under OGD/R condition (*P* < 0.05; Figures 8A–E, 9A–D), suggesting the reversal of osmotin effect on proinflammatory factors expression. However, osmotin + LY294002 administration affected anti-inflammatory factors resulted in the opposite impacts, that osmotin-induced upregulations of IL-4, IL-10, and IL-13 were all recovered by addition of osmotin + LY294002 (Figure 9E). All these data suggested that PI3K/AKT pathway might play an important role in the protective effect of osmotin on H9c2 cells after OGD/R-induced injury. Osmotin might have anti-inflammatory effects on OGD/R-induced injury in H9C2 cells by activating of AdipoR1/PI3K/AKT pathway.

## Discussion

In this study, our findings suggested that the osmotin, as an adiponectin homolog, might protect H9c2 cells against I/R *in vitro*. This protective effect was mainly related with anti-inflammatory and anti-apoptosis properties, and also might be with receptor dependent. In addition, we find that the AdipoR1 receptor was related with activation of PI3K/AKT, leading to the inhibition effect on cell apoptosis and inflammatory response. Osmotin is a multifunctional plant protein which derived from tobacco. In addition to the protective effect of osmotin on plants, osmotin has been also revealed to have neuroprotective effect (Shah et al., 2014, 2016; Ullah et al., 2014; Ali et al., 2015; Badshah et al., 2016). Recently, was confirmed that osmotin had structural and functional similarity compare with human adiponectin (Narasimhan et al., 2005; Miele et al., 2011). Several studies confirmed that osmotin had the same protective function as adiponectin due to the similar stucture (Naseer et al., 2014; Shah et al., 2014; Badshah et al., 2016). In this study, our findings suggested that the osmotin might be able to protect H9c2 cells against I/R induced injury *in vitro*.

Osmotin is an adiponectin homolog, and adiponectin is a cytokine which was primarily generated by adipocytes with insulin-sensitizing, anti-inflammatory and anti-oxidant properties (Kadowaki et al., 2006; Oh et al., 2007; Chan et al., 2012). Adiponectin is mainly through two receptors AdipoR1 and AdipoR2 to play its function. It had been reported that adiponectin and it receptors were expressed in human and murine cardiomyocytes (Piñeiro et al., 2005; Ding et al., 2007), and play significant roles in various cardiovascular diseases (Maia-fernandes et al., 2008; Wang et al., 2009; Villarreal-Molina and Antuna-Puente, 2012), including protecting against myocardial I/R injury (Tao et al., 2007; Wang et al., 2010; Liu et al., 2011). Based on these functions of adiponectin and its receptors, we assumed that osmotin might also have cardioprotective effect via modulating AdipoRs. In our study, the H9c2 cells which were used as simulate myocardial I/R injury model was administrated with osmotin, and as expected, the cell viability was significantly decreased by OGD/R. While osmotin increased cell viability, suggesting the cardioprotective effect of osmotin. Even when AdipoR1 and AdipoR2 were silenced in pretreated H9c2 cells, respectively. Cell viability was only suppressed by AdipoR1 silence but not AdipoR2 compared to administration of osmotin alone. Therefore, protective effect of osmotin on I/R induced H9c2 cells might be via AdipoR1.

It has been well acknowledged that the cell apoptosis of cardiomyocytes in cardiac injury is a continuing dynamic process which is related with I/R. Reduction of cell apoptosis is believed to be an effective therapeutic strategy for treatment of myocardial I/R injury. The effects of osmotin on cell apoptosis and the expression of inflammatory factors in H9c2 cells were further verified. We found that osmotin showed anti-apoptosis property that reduced H9c2 cell apoptosis after OGD/R injury which was in line with previous studies (Narasimhan et al., 2005; Naseer et al., 2014; Shah et al., 2014). These results suggested effects of osmotin on apoptotic related factors as the increased levels of anti-apoptotic family member Bcl-2 and inactive form of caspase-3 (pro-caspase-3), while the decreased levels of the proapoptotic family member Bax and cleaved caspase-3 compared with vehicle group. All these results suggested that the influence of osmotin on the levels of the Bcl-2 and caspase family proteins might be a significant component of its protective action for H9c2 cells.

In addition to the cell apoptosis, I/R can also lead to acute inflammatory response which might cause substantial cellular injury and organ dysfunction (Frangogiannis et al., 2002; Oyama et al., 2004). In this study we also assessed LDH and ROS release activity about I/R induced H9c2. Results suggested that osmotin suppressed LDH and ROS release activity of I/R injured cells, while AdipoR1 knockdown revised LDH and ROS release, suggesting after simulated I/R injury, osmotin might relieve cell damage via AdipoR1. The inflammatory response following I/R is believed to be involved in activation of transcription factor NF-κB, which stimulates several inflammatory mediators expressions including TNF-α, IL-1β, IL-6, and IL-8 (Medzhitov et al., 1997; Frantz et al., 1999; Baumgarten, 2001). Thus, limitation of these inflammatory mediators might be helpful to the treatment of myocardial I/R. As results of this study, we found that in simulated I/R injury of H9c2 cells, the expressions of proinflammatory factors inluding TNF-α, IL-1β, IL-6, and IL-8 were all dramatically decreased, while anti-inflammatory factors including IL-4, IL-10, and IL-13 were all increased after osmotin treatment, suggesting that osmotin was involved in reducing the inflammatory response. This result was similar with a previous study that osmotin could attenuate lipopolysaccharide (LPS)-induced neuro-inflammation (Badshah et al., 2016). Nevertheless, our results also showed that all these effects of osmotin were reversed by knockdown of AdipoR1 in cells, suggesting that the protective function of osmotin on H9c2 cells was related with AdipoR1. Thus, the potential inhibitory inflammatory response of osmotin might contribute to the protective function of osmotin in I/R injury via AdipoR1.

It is well known that the PI3K/AKT pathway has significant biological functions in cell proliferation, survival apoptosis and inflammatory response (Cantley, 2002). Previous studies have revealed that activation of PI3K/AKT pathway improved cardiac contractility, alleviated inflammatory response, declined cardiomyocyte apoptosis, and thus ameliorated myocardial I/R injury (Meijing et al., 2009; Fang et al., 2011; Arslan et al., 2013). In the present study, we observed that osmotin significantly increased the phosphorylation levels of PI3K and AKT in H9c2 cells after simulated I/R injury, suggesting that osmotin might be related with activation the PI3K/AKT pathway. However, these effects were reduced by knockdown of AdipoR1. To further confirm the relationship between protective function of osmotin and PI3K/AKT pathway, we analyzed the cell viability, cell apoptosis, and the expression of inflammatory factors again after administration of the inhibitor of PI3K (LY294002). The results showed that the effects of osmotin on the cell viability, apoptosis, and the expression of inflammatory factors were reversed by administration of LY294002. Combined with the above results, it suggested that the induced activation of PI3K/AKT and inhibited NF-κB resulted in the inhibition effect on cell apoptosis and expression of inflammatory cytokines, eventually protected H9c2 cells against I/R injury.

There existed several limitations in the present study. Firstly, in this study H9c2 cell line was used to establish an *in vitro* I/R injury model. However, H9c2 cells do not display mature sarcomeric organization and possess β-tubulin II, a mitochondrial isoform of tubulin that plays an important role in mitochondrial function and regulation and may contribute to the decreased cell viability of H9c2 cells in response to I/R injury compared to other lines of cardiomyocytes (Kuznetsov et al., 2015). Thus, more efforts are required to explore whether the protective functions of osmotin can be reproduced in primary cardiomyocytes. Secondly, RISK and SAFE (JNK/STAT3) are also two main cardioprotective molecular pathways (Santos-Gallego et al., 2016). This study includes the lack of investigations regarding whether osmotin exerted cardioprotective functions also via these two pathways. Thirdly, we did not add the treatment with adiponectin as a positive control, which may better reveal the adiponectin-like effect of osmotin on myocardial I/R. Fourthly, we focused on the *in vitro* cardioprotective effects of osmotin, *in vivo* investigations will largely improve the findings in the present study. In conclusion, osmotin had a significant protective effect on I/R injury of H9c2 cells. This protective effect was mainly related to anti-inflammatory and anti-apoptosis properties through the AdipoR1/PI3K/AKT signaling pathway.

## Author contributions

JL: experimental design; perform the experiments; collected the data; data interpretation; wrote the manuscript. HS: perform the experiments; collected the data; data interpretation; wrote the manuscript. JZ: perform the experiments; collected the data; data interpretation; wrote the manuscript. YW: perform the experiments; collected the data; wrote the manuscript.

### Conflict of interest statement

The authors declare that the research was conducted in the absence of any commercial or financial relationships that could be construed as a potential conflict of interest.

## Supplementary material

Supplementary Figure 1 Effects of osmotin H9c2 cell viability. **(A)** Effects of various dose of osmotin on the cell viability was detected by MTT assay (*n* = 5). **(B)** Effects of various dose of osmotin on the cell viability under OGD/R stimulating conditions was detected by MTT assay (*n* = 5). Different letters above the columns indicate that the means of different groups were significantly different (*P* < 0.05) by ANOVA. MTT, 3-(4, 5-dimethylthiazol-yl)-2, 5-diphenyl-2-H-tetrazolium bromide; OGD/R, oxygen and glucose deprivation/reperfusion.

Supplementary Table 1 Sequences of the siRNAs.

Supplementary Table 2 Sequences of PCR primers.
