# Supplementary material for: Osmotin Protects H9c2 Cells from Simulated Ischemia-Reperfusion Injury through AdipoR1/PI3K/AKT Signaling Pathway
Source: Front Physiol. 2017 Sep 25;8:611. doi: 10.3389/fphys.2017.00611 (PMC5622187; doi:10.3389/fphys.2017.00611)
Supplement: Supplementary Figure 1 — Effects of osmotin H9c2 cell viability. (A) Effects of various dose of osmotin on the cell viability was detected by MTT assay (n = 5). (B) Effects of various dose of osmotin on the cell viability under OGD/R stimulating conditions was detected by MTT assay (n = 5). Different letters above the columns indicate that the means of different groups were significantly different (P < 0.05) by ANOVA. MTT, 3-(4, 5-dimethylthiazol-yl)-2, 5-diphenyl-2-H-tetrazolium bromide; OGD/R, oxygen and glucose deprivation/reperfusion. [file Image1.PDF]

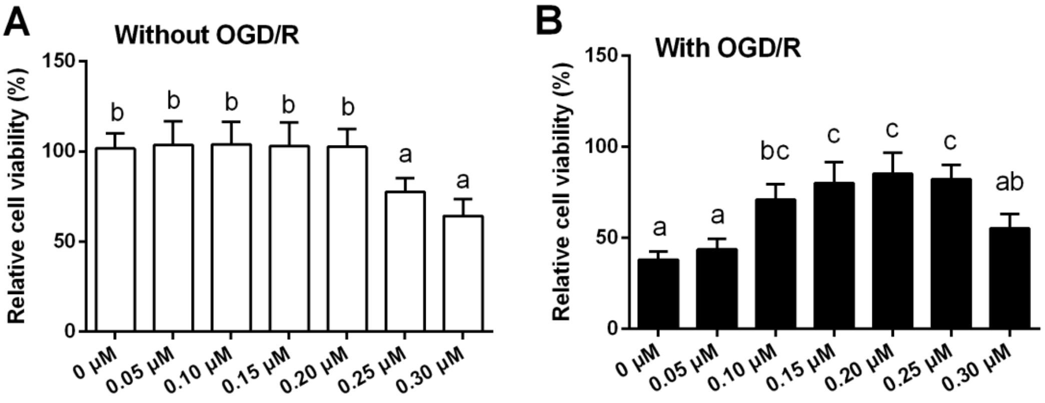


**Supplementary Figure 1** Effects of osmotin H9c2 cell viability. A. Effects of various dose of osmotin on the cell viability was detected by MTT assay (n=5). B. Effects of various dose of osmotin on the cell viability under OGD/R stimulating conditions was detected by MTT assay (n=5). Different letters above the columns indicate that the means of different groups were significantly different ($P$ < 0.05) by ANOVA. MTT, 3-(4, 5-dimethylthiazol-yl)-2, 5-diphenyl-2-H-tetrazolium bromide; OGD/R, oxygen and glucose deprivation/reperfusion;
