# Supplementary material for: Osmotin Protects H9c2 Cells from Simulated Ischemia-Reperfusion Injury through AdipoR1/PI3K/AKT Signaling Pathway
Source: Front Physiol. 2017 Sep 25;8:611. doi: 10.3389/fphys.2017.00611 (PMC5622187; doi:10.3389/fphys.2017.00611)
Supplement: Supplementary Table 1 — Sequences of the siRNAs. [file Table1.DOCX]

**Supplementary Table 1** Sequences of the siRNAs

|  | Sequences |
| --- | --- |
| Rat AdipoR1 siRNA | 5′-UACAACACCACUCAAGCCAAGUCCC-3’ |
| Rat AdipoR2 siRNA | 5′-AACAGGUGUCUCUAAACUGGGCUCC-3’ |
| Rat siRNA control (NC) | 5′-AUUUAACUUCUGGUGACGAUACUGG-3’ |
