# Supplementary material for: Osmotin Protects H9c2 Cells from Simulated Ischemia-Reperfusion Injury through AdipoR1/PI3K/AKT Signaling Pathway
Source: Front Physiol. 2017 Sep 25;8:611. doi: 10.3389/fphys.2017.00611 (PMC5622187; doi:10.3389/fphys.2017.00611)
Supplement: Supplementary Table 2 — Sequences of PCR primers. [file Table2.DOCX]

**Supplementary Table 2** Sequences of PCR primers

| Gene | Sense | Anti-sense | Accession number |
| --- | --- | --- | --- |
| *AdipoR1* | 5’-CCAGGAAGA  AGAGGAGGAC-3’ | 5’-ATGTAGCAGAT  AGTCGTTGTC-3’ | NM_00120629.1 |
| *AdipoR2* | 5’-TTCTTTCTTCT  CCCTTTCTCTC-3’ | 5’-CTCCCTCCC  TCCCTTACC-3’ | NM_024551.2 |
| *GAPDH* | 5’-CCACATCGCTCA GACACCAT-3’ | 5’-ACCAGGCGCCCA  ATACG-3’ | NM_001289745.1 |
